# A content analysis of YouTube videos on tinnitus in South Korea

**DOI:** 10.1038/s41598-023-40523-9

**Published:** 2023-08-21

**Authors:** Hee Won Seo, Jung Woo Ha, Jin Hye Kwak, Moo Keon Kim, Hayoung Byun, Seung Hwan Lee, Jae Ho Chung

**Affiliations:** 1 Department of Otolaryngology-Head and Neck Surgery, College of Medicine, Hanyang University, Seoul, Republic of Korea; 2 College of Medicine, Hanyang University, Seoul, Republic of Korea

**Keywords:** Auditory system, Patient education, Health services

## Abstract

More people use the internet for medical information, especially YouTube. Nevertheless, no study has been conducted to analyze the quality of YouTube videos about tinnitus in Korea. This study aims to review the contents and quality of YouTube videos on tinnitus. The top 100 Korean YouTube videos on tinnitus were reviewed by a tinnitus expert. This study assessed video details: title, creator, length, and popularity indicators—subscribers, views, and likes. The contents of the video clips were analyzed to determine the relevance, understandability, actionability, and quality of information. Out of 100 tinnitus videos, 27 were created by otolaryngologists, 25 by traditional Korean medicine doctors, 25 by other medical professionals, and 3 by lay persons. Sensorineural tinnitus was frequently dealt, and hearing loss, stress, and noise were introduced as main causes of tinnitus. Otolaryngologists' videos covered verified treatments, but others suggested unproven therapies including herbal medicine or acupressure. Otolaryngologists' videos showed significantly higher understandability and quality of information compared to others (p < 0.001). This study found that tinnitus YouTube videos frequently present low-quality and incorrect material, which could have an adverse effect on patients. Results highlight the need for tinnitus specialists to provide accurate information.

## Introduction

Tinnitus is a common and distressing auditory symptom that affects individuals across various age groups and populations. It is characterized by the perception of sound in the absence of an external stimulus, and it can significantly impact an individual's quality of life.^1,2^

The prevalence of tinnitus varies across different populations and is affected by various factors such as age, gender, and hearing status. A meta-analysis of 23 studies reported a prevalence of approximately 10% in the general population^3^. And a population based survey of 1609 participants in South Korea found that the prevalence of tinnitus was 20.7%^4^. Moreover, the prevalence of tinnitus is higher among older individuals, with up to 30% of those over the age of 65 experiencing tinnitus. Furthermore, tinnitus can be a significant economic burden on society^5^. An increase in social awareness of tinnitus may lead to a greater demand for medical information for tinnitus, prompting the development of more accurate and accessible sources of medical information for tinnitus.

The recent increase in internet usage has made medical information more accessible to individuals with tinnitus, with You Tube emerging as a popular and convenient source for obtaining medical information. YouTube is a video-based social media platform that has gained tremendous popularity due to its open environment that allows interactive communication, and is currently ranked as the second most commonly accessed website with an estimated 30 million users accessing it on a daily basis^6^. In South Korea as well, according to an update from Google's advertising resources, as of early 2023, the number of YouTube users in South Korea was estimated at 46 million, representing 88.8% of the total population. A recent systematic review postulated that YouTube has emerged as a platform for promoting unscientific therapies and drugs that lack approval from appropriate regulatory process^7,8^. Likewise, although the number of YouTube videos about tinnitus has increased significantly, the reliability and accuracy of these videos have not been thoroughly evaluated, indicating a need for careful assessment of the information presented by YouTube.

Notably, in South Korea, traditional Korean medicine plays a unique role in the healthcare. Traditional Korean medicine doctors recognized as healthcare professionals who can independently diagnose and treat patients. Unlike physicians who possess specialized knowledge and training to conduct evidence-based treatments, traditional Korean medicine doctors often introduce therapies such as herbal medicine, acupressure, and acupuncture, which may lack robust scientific evidence. Given the characteristic of YouTube where content can be freely produced by anyone, videos about tinnitus can be created and uploaded by otolaryngologists, traditional Korean medicine doctors, or even non-medical personnel. This diversity in content creators has the potential to lead to a wide range of information quality, potentially complicating the search for reliable information for individuals with tinnitus.

As public interest in tinnitus continues to rise, so does the importance of ensuring the accuracy and credibility of online information sources. The overflow of information can make it difficult for patients to find proper treatment for tinnitus, leading to anxiety and confusion. Therefore, a more precise evaluation of medical information about tinnitus is needed to support patients in finding appropriate treatment methods. Therefore, it was deemed necessary to thoroughly evaluate the quality of YouTube videos related to tinnitus from the perspective of evidence-based medicine.

The aim of the present study was to comprehensively evaluate the quality of YouTube videos related to tinnitus, with a focus on assessing factors such as the credibility of the creator, the accuracy and relevance of the content, and the understandability, actionability, and information quality of the video clips.

## Methods

### Video search

The present study conducted content analysis on the top 100 videos retrieved via search for the Korean translations of the keywords of "tinnitus" (이명, pronounced "ee-myung") and "tinnitus treatment" (이명 치료, pronounced "ee-myung chi-ryo") on the YouTube platform as of January 30th, 2023. The choice of 100 videos was guided by practical considerations and the desire to include the most viewed and potentially influential videos on the subject of tinnitus in our sample. When we extended our search beyond the top 100 videos, we observed a noticeable drop in views, often falling below 3000 views, and the channels hosting these videos typically had fewer subscribers, implying less overall influence. This decision is also supported by the observation that most users tend to review only the first few pages of search results^9,10^. To eliminate the potential bias introduced by YouTube's personalization algorithm, the search was performed using a new account with no prior search history. The search was restricted to YouTube videos that were produced in the Korean language, with those providing sound therapy (eg. White noise, pink noise, etc.) for tinnitus treatment being excluded from consideration.

### Metadata of tinnitus video clips

After the video selection process, an otolaryngologist was assigned the task of evaluating and analyzing the content information presented in the selected video clips. Basic descriptive data were collected and reviewed for each video, including information such as the video's title, video length, total number of views, as well as the number of “Likes” and “Subscribers” associated with each video clip. For the assessment of the creator, the present study grouped the creators of the YouTube video clips into different categories based on their professions. The professions were determined primarily based on the account descriptions, with additional verification done where possible. In instances where we were unable to definitively determine the profession, the video was classified as 'unknown.' These categories included otolaryngologists, traditional Korean medicine doctors, medical professionals other than otolaryngologists, lay persons, and unknown.

### Contents analysis

The video content was also evaluated by an otolaryngologist whether the content of the video clip covers tinnitus diagnosis and tinnitus treatments based on the otology textbook. Regarding the diagnosis, we classified whether the subjective tinnitus, namely, the mention of subjective perception of sound, and objective tinnitus, the mention of sound that can be heard by others, were identified. Additionally, we investigated whether the causes of tinnitus such as noise, aging, acoustic neuroma, hearing loss, vascular issues, myoclonus, and stress were addressed. With respect to treatment methods, we examined whether tinnitus retraining therapy, including sound therapy, medication, neuromodulation, cognitive-behavioral therapy, hearing aids, and cochlear implants were mentioned. Furthermore, we investigated the purpose of the production of the video clips, whether it was for patient education, personal or hospital promotion, or for the sale of related products.

### Assessment of understandability, actionability, and information quality

In order to evaluate the understandability, actionability, and the quality of the information provided in the videos, specific assessment tools were utilized.

For the understandability and actionability, the Patient Education Materials Assessment Tool for Audio and Visual patients’ information (PEMAT-A/V) was employed. This is a widely used health literacy assessment method that can be employed to evaluate patient education materials developed by the Agency for Healthcare Research and Quality^11^. The tool, which consists of 17 items related to understandability and 4 items related to actionability, has strong internal consistency, reliability, and construct validity. The scoring method of PEMAT-A/V involves rating the materials on a scale from 0 to 100% for both the understandability and actionability dimensions. The ratings are based on a set of objective and subjective criteria that are designed to capture the overall effectiveness of the materials in conveying information and promoting behavior change (Table 1). The scoring method involves assigning a score of 1 for agreement, 0 for disagreement, and no score for items deemed not applicable. After scoring each item, the total points are summed for each subscale (understandability and actionability) and divided by the total possible points. The resulting score is then multiplied by 100 to obtain a percentage score for each subscale. Higher percentages indicate higher levels of understandability and actionability, while scores below 70% indicate poor understandability or actionability of the information being assessed.

**Table 1:** Patients education materials assessment tool for audiovisual materials (PEMAT-A/V) score for understandability and actionability.

Understandability	
Topic: Content	
1	The material makes its purpose completely evident
Topic: Word choice and style	
3	The material uses common, everyday language
4	Medical terms are used only to familiarize audience with the terms. When used, medical terms are defined
5	The material uses the active voice
Topic: Organization	
8	The material breaks or “chunks” information into short sections
9	The material’s sections have informative headers
10	The material presents information in a logical sequence
11	The material provides a summary
Topic: Layout and design	
12	The material uses visual cues (e.g., arrows, boxes, bullets, bold, larger font, highlighting) to draw attention to key points
13	Text on the screen is easy to read
14	The material allows the user to hear the words clearly (e.g., not too fast, not garbled)
Topic: Use of visual aids	
18	The material uses illustrations and photographs that are clear and uncluttered
19	The material uses simple tables with short and clear row column headings
Actionability	
20	The material clearly identifies at least one action the user can take
21	The material addresses the user directly when describing actions
22	The material breaks down any action into manageable, explicit steps
25	The material explains how to use the charts, graphs, tables, or diagrams to take actions

For the assessment of information quality and reliability, a modified DISCERN score was utilized. The DISCERN is a standardized tool for evaluating the quality of health information, and the modified DISCERN index is used to evaluate reliability and accuracy using a five-point scale. Each point is allocated for concision, reliability, balance, reference, and uncertainty (Table 2). This tool was previously utilized in several studies for the same purpose^9,12,13^. The score comprises five yes/no questions, with 1 point given for each "yes" answer and 0 points for "no". The maximum total score is 5, with higher scores indicating better quality and reliability of the information. To evaluate the appropriateness of each point, we referenced the clinical practice guidelines for tinnitus published by the American Academy of Otolaryngology–Head and Neck Surgery (AAO-HNS) in 2014^14^ and the tinnitus chapters (Chapter 52–54) in the 2018 2nd revised edition of the Korean Textbook of Otorhinolaryngology^15^.

**Table 2:** Modified DISCERN score for quality assessment.

Modified DISCERN description	
1	Are the video’s aims clear, concise, and achieved?
2	Are valid and reliable sources cited?
3	Is the information discussed balanced and unbiased?
4	Are additional sources of information listed for patient reference?
5	Does the video address areas of controversy and uncertainty?

### Statistics

All statistical analyses were done using SPSS 27.0 (IBM, Armonk, NY). Descriptive data of number of Likes, views or subscribers are expressed as means and standard deviations. χ^2^ tests for categorical variables, and two-tailed Student’s t-tests were used for continuous variables of understandability, actionability and information quality scores. The Pearson correlation test was used to assess the relation between number of views and assessment scores. A *P* value < 0.05 was considered statistically significant.

### Ethics

The current study was waived from approval by Institutional Review Board (IRB) of Hanyang University Hospital as it did not involve the medical data or patients’ information.

## Results

### General information for video clips on tinnitus

The present study examined 100 of the most frequently viewed videos on tinnitus. Of these, 27 were produced by otolaryngologists, 25 were created by traditional Korean medicine doctors, and 25 were produced by other medical professionals including dentists, psychiatrists, neurologists, family physicians, and physiotherapists. Additionally, 3 videos were created by members of the public (Table 3).

**Table 3:** Profession of video creators for top 100 You-Tube video clips on tinnitus.

Professions	Total (N = 100)
Otolaryngologists	27
Traditional Korean medicine Doctors	25
Other medical professionals	25
Dentists	12
Psychiatrists	6
Neurologists	2
Integrative medicine	2
Pharmacists	2
Physical therapist	1
Family medicine doctors	1
Lay persons	3
Nutritionist	2
Natural therapist	1
Unknown	20

Among the 100 most viewed videos, the video clip with the highest number of views was created by traditional Korean medicine doctors, with a total of 1,540,000 views. This video introduced acupressure techniques for tinnitus treatment. Out of the top 10 videos, 4 were created by traditional Korean medicine doctors, 2 were created by otolaryngologists, 1 was created by a dentist, 1 was created by an integrative medicine practitioner, and 2 were created by the lay persons. (Table 4).

**Table 4:** Top 10 You-Tube video clips on tinnitus.

Rank	Video name	Number of views	Name of creator	Profession of video producer
1	Simple acupressure method on tinnitus	1,540,000	Heo Jun Grandma Health TV	Traditional Korean medicine doctor
2	If you've ever heard a beep in your ears, look!	1,430,000	Doctor Friends	Otolaryngologist
3	If you really want to treat tinnitus, 'this' ~ the easiest way	1,010,000	Heo Jun Grandma Health TV	Traditional Korean medicine doctor
4	Tinnitus, beeping in the ears, it will go away if you manage it like this	800,000	Dried herbs tv	Lay persons
5	An acupuncture point that makes tinnitus disappear when you tap it	780,000	Soonyeol Kim tv	Traditional Korean medicine doctor
6	Why do I get Ears Ringing	720,000	Integrative Medicine	Integrative Medicine
7	Press here for 1 min! The beep in the ears, the tinnitus completely disappears	650,000	Sky Health	Lay persons
8	Acupoints for improving ringing in the ears	520,000	Kim Sohyung Channe	Traditional Korean medicine doctor
9	Dizziness and Tinnitus	450,000	Louisbin Dental	Dentist
10	'Tinnitus', a problem of the cerebrum, not the cochlea"	420,000	Yunhap news	Otolaryngologist

Table 5 showed the descriptive statistics for the number of subscribers, number of views, likes, and video length (duration) of these top 100 videoclips. Mean number of views was 142,156 ranging from 3400 to 1,540,000.

**Table 5:** Statistics of meta-data in 100 most viewed video clips on tinnitus.

	Mean ± SD	Minimum	Maximum
Total			
Number of subscribers (number)	247,016.0 ± 466,019.9	163	3,140,000
Number of views (number)	142,156 ± 271,856	3400	1,540,000
Number of likes (number)	2400 ± 5088	16	33,000
Video length (second)	663 ± 565	57	3106

### Contents analysis

Table 6 presents the frequency analysis of mentioned topics in the top 100 videos, comparing the proportion of mentioned elements in videos created by otolaryngologists with those created by other professionals or lay persons. Regarding diagnosis of tinnitus, 66% of videos dealt with sensorineural tinnitus while objective tinnitus was mentioned only 22% of top 100 video clips. Otolaryngologists are more frequently mentioned both sensorineural and objective tinnitus than other creators regarding tinnitus diagnosis.

**Table 6:** Contents analysis on 100 most viewed video clips on tinnitus.

Mentioned contents	Frequency count			p-value^§^
	Total (n = 100)	Otolaryngologist (n = 27)	Others^a^ (n = 73)	
Diagnosis				
Sensorineural tinnitus	66	25 (92.6%)	41 (56.2%)	0.001
Objective tinnitus	22	12 (44.5%)	10 (13.7%)	0.007
Cause of tinnitus				
Noise	25	11 (40.7%)	14 (19.2%)	0.027
Hearing loss	36	21 (77.8%)	15 (20.5%)	< 0.001
Acoustic tumor	7	4 (14.8%)	3 (4.1%)	0.083
Vascular problem	15	10 (37.0%)	5 (6.8%)	0.01
Myoclonus	9	5 (18.5%)	4 (9.6%)	0.057
Aging	10	3 (11.1%)	7 (9.6%)	0.822
Stress	29	7 (25.9%)	22 (30.1%)	0.806
Medication	4	2 (7.4%)	2 (2.7%)	0.297
Other cause of tinnitus^b^	47	2(7.4%)	45(61.6%)	< 0.001
Treatment of tinnitus				
Tinnitus retraining therapy	17	14 (51.9%)	3 (4.1%)	< 0.001
Medication	17	13 (48.15)	4 (5.5%)	< 0.001
Hearing aids	16	14 (51.9%)	2 (2.7%)	< 0.001
Neuromodulation	12	10 (37.0%)	2 (2.7%)	< 0.001
Cognitive behavioral therapy	16	16 (59.3%)	0	< 0.001
Others treatment methods^c^	77	4 (14.8%)	73 (100%)	< 0.001
Purpose of YouTube video				
Education	79	26 (96.3%)	53 (72.6%)	0.010
Sharing personal experience	7	0	7 (9.6%)	0.095
Advertisement	61	6 (22.2%)	55 (75.3%)	< 0.001

^a^Others: Traditional Korean medicine doctors, other medical professionals, lay persons, and unknown.

^b^Sinusitis, Rhinitis, Otitis media, sleep apnea, Eustachian dysfunction, Liver disorders, diet habit, excessive weight loss or exercise, vascular problem, Hormonal imbalance, Intoxication, Renal dysfunction, Hypothyroidism, general myalgia, etc.

^c^Middle ear implant, Bone anchored hearing aid, Acupuncture, moxibustion, Acupressure, Nerve Block, Food therapy, Nutrition support, Vitamin, Herbal medicine, Chiropractic, Massage, manual therapy, hydration, intake minerals, etc.

^§^Chi-Square test.

Hearing loss, noise, vascular problem, and stress were frequently mentioned as cause of tinnitus. The videos created by otolaryngologists emphasized the importance of hearing loss and noise exposure, while other video creators frequently mentioned stress and various factors including dietary habits, cervical problems, kidney or liver dysfunction, thyroid disorders, metabolic imbalances, toxicity and heavy metal poisoning, manganese deficiency, lack of exercise, and nutritional imbalances as potential causes of tinnitus.

Regarding treatment of tinnitus, Tinnitus retraining therapy, medication, hearing aids, and neuromodulation were mentioned in the video by otolaryngologists, other creators delt with acupuncture, nerve block, diet therapy, exercise, food therapy, relieving stress and modification of sleep style, etc. In addition, the purpose of tinnitus videos created by otolaryngologists was primarily for patient education, accounting for 96% of the videos analyzed. However, while a considerable portion (72.6%) of videos created by other producers were also primarily intended for educational purposes, it should be noted that an even greater proportion (75.3%) were produced with promotional or advertising intentions. This demonstrates a significant difference in content creation objectives compared to otolaryngologists.

### Understandability, actionability, and information quality

**Figure 1:**
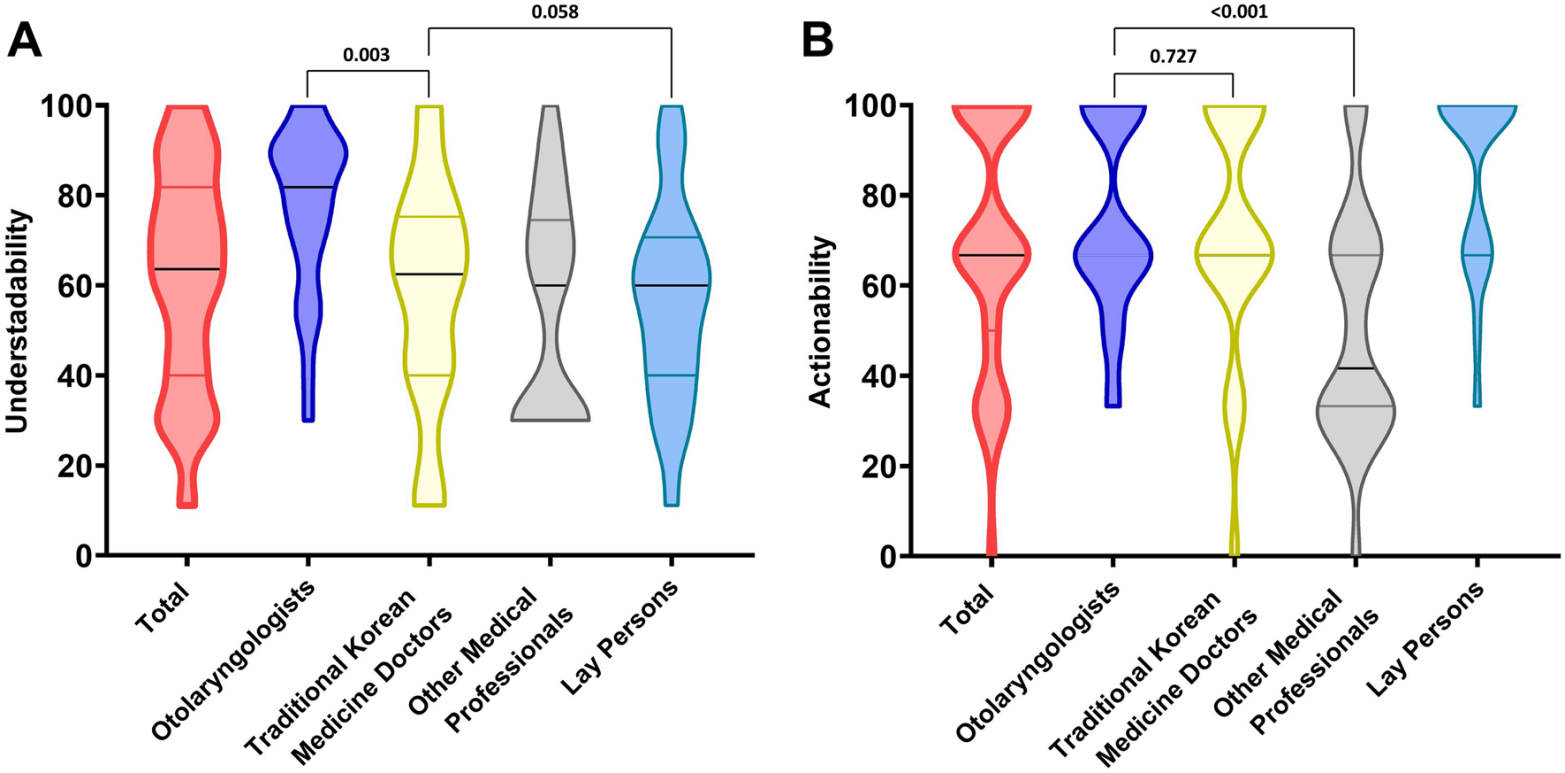
Violin plots of Understandability and Actionability scores for tinnitus video clips according to the profession of the creators. (**A**) Distribution of understandability score, (**B**) distribution of actionability score.

Mean PEMAT-A/V scores for understandability and actionability were 62.0 ± 24.4 and 70.4 ± 26.6, respectively (Table 7). The videos created by otolaryngologists had a significantly higher understandability score compared to those created by other medical professionals (p < 0.001). Figure 1 showed the PEMAT-A/V scores according to the video creators, video from otolaryngologists showed significantly higher score of understandabilities than those from traditional Korean medicine doctors (Fig. 1A). In terms of actionability, score of otolaryngologists’ videos was significantly higher in those of other medical professionals (p < 0.001, Fig. 1B). According to correlation analysis, both understandability and actionability were not significantly correlated with number of views (p = 0.980 and p = 0.836 respectively).

**Table 7:** Assessment of understandability, actionability, and information quality for tinnitus video clips using patients education materials assessment tool for audiovisual materials (PEMAT-A/V) and modified DISCERN score.

	Total	Otolaryngologist (n = 27)	Others^a^ (n = 73)	p-value^†^
PEMAT-A/V score				
Understandability score	62.0 ± 24.4	77.1 ± 18.5	56.4 ± 24.0	< 0.001
Actionability score	70.4 ± 26.6	74.7 ± 21.9	68.8 ± 28.4	0.335
Modified DISCERN score	2.04 ± 1.55	3.78 ± 0.93	1.40 ± 1.20	< 0.001

^a^Others (Traditional Korean medicine doctors, other medical professionals, lay persons, and unknown).

^†^T-test.

For the quality of information as assessed by the modified DISCERN score, the mean score for otolaryngologists was 3.78 ± 0.93, for traditional Korean medicine doctors 1.28 ± 0.98, and for other medical professionals 1.28 ± 1.37. When compared with others (1.40 ± 1.20), otolaryngologists' videos demonstrated a higher level of reliability (p < 0.001, Table 7). No significant correlation was found between the modified DISCERN score and the number of views (p = 0.946).

## Discussions

The present study reviewed the top 100 most viewed YouTube videos on tinnitus and tinnitus management in South Korea and conducted the contents analysis. Videos related to tinnitus were created by otolaryngologists, traditional Korean medicine doctors, and other medical professionals such as dentists, psychiatrists, neurologists, family physicians, audiologists, and physiotherapists. Among these, 27 videos were found to have been created by otolaryngologists.

The content of the videos showed a higher proportion of non-otolaryngologic explanations, such as those from traditional Korean medicine and other non-medical sources. Most of tinnitus videos were created for patient education and promotion purposes. For the last, videos created by otolaryngologists were found to have a higher level of understandability compared to those created by other professionals. To our knowledge, this work is the first to evaluate the YouTube video clips on tinnitus distributed in South Korea and concluded that substantial number of YouTube contents about tinnitus were based on inappropriate medical evidence and had poor educational quality.

Various medical information is being provided through YouTube videos, and patients can easily access this video clips. In the otolaryngology fields, the educational benefit of YouTube videos about cholesteatoma, tonsillectomy, ventilation tubes for serous otitis media, rhinoplasty, and thyroid cancer had been examined^6,8,10,16–18^. A recent study analyzing YouTube videos related to cholesteatoma revealed that the educational level of these videos was extremely low, and the content was reported to lack evidence-based information^8^. Regarding pediatric tonsillectomy, there was a range of information available from highly valuable to unhelpful or misleading^19,20^. It is noteworthy that anecdotal evidence of unproven alternative therapies and biased negative reporting of medically proven therapies are widely available to patients with YouTube videos, and if they rely solely on this information to make decisions on treatment, significant safety consequences may ensue^7,8^. And previous studies also postulated that physicians need to be aware of the possible impact these video websites may have on patient perceptions^19,20^. The findings of the present study showed that tinnitus video uploaded by traditional Korean medicine practitioners or physicians other than otolaryngologists dealt with scientifically unproven treatments of acupressure, acupuncture, moxibustion, nerve block, Herbal medicine, Chiropractic, manual therapy, etc.

In South Korea, tinnitus is a prevalent condition, and both otolaryngologists and traditional Korean medicine doctors are involved in tinnitus management^21^. Otolaryngologists use a variety of treatments for tinnitus, including tinnitus retraining therapy, medication, neuromodulation, and hearing aids. Tinnitus retraining therapy involves directive counselling and sound therapy to habituate the patient to the sound of their tinnitus. Neuromodulation techniques, such as transcranial magnetic stimulation and vagus nerve stimulation, are also used to manage tinnitus. Medications of antidepressants or anticonvulsants, are used to alleviate symptoms accompanied with tinnitus and hearing rehabilitation can be used to amplify external sounds and reduce the perception of tinnitus^22^. On the other hands, traditional Korean medicine doctors use traditional methods such as acupuncture or moxibustion. With such a wide variety of treatments available, it's critical to refer to evidence-based guidelines to discern their respective efficacy. According to the 2014 AAO-HNS clinical practice guideline, tinnitus retraining therapy and hearing aids are explicitly recommended as effective treatments for tinnitus management, as their efficacy has been validated through comprehensive clinical trials. Neuromodulation techniques and medical therapy are regarded as potential options depending on individual patient needs, but with a cautionary note on their varying levels of scientific evidence supporting their use. Contrastingly, traditional methods such as acupuncture do not receive the same level of endorsement. Acupuncture, in fact, is identified as having 'no recommendation' due to its lack of supporting evidence. Other traditional therapies, as practiced by traditional Korean medicine doctors, are not even mentioned in the guideline, reflecting their questionable efficacy^14^. So, the effectiveness of these those traditional treatments on tinnitus remains unclear^23^. Tinnitus also has been the subject of YouTube videos created by dentists, neurologists, psychiatrists, integrative medicine, pharmacists, family medicine doctors, nutritionist etc. Their videos offer a wide range of explanations for the causes of tinnitus and introduce treatment approaches not typically discussed in otolaryngology including nerve block, food therapy, nutrition support, vitamin intake, manual therapy, etc. Those therapies are medically unproven, so tinnitus patients should be cautious when relying on YouTube videos as a primary source of information.

Tinnitus has diverse causes and complicated treatment methods, making it difficult to achieve complete cure with existing treatments^24^. In such situations, patients often fail to respond to treatment or become anxious, leading to increased demand for medically unproven therapies. In addition, in an environment where anyone can publish and share medical information through social media including YouTube, both experts and non-experts can easily express their opinions. In this situation, videos created by traditional Korean medicine doctors or non-medical professionals often present themselves as easy and quick solutions, which could explain their high viewership among the general public. Thus, it is necessary to verify such unreliable information because it may lead to misunderstanding of tinnitus patients. Similar results were identified in a research investigation that looked at YouTube tinnitus videos in the English language, with most of the videos consisted of patient experiences^25^. To guarantee that reliable information is given to patients, the previous study emphasizes the necessity for medical professionals to make efforts to deliver information validated by medical evidence^25^.

PEMAT-A/V is a reliable and valid instrument to evaluate the understandability and actionability of audiovisual patient education materials^11^. The strong internal-consistency and inter-rater reliability of this tool provide a robust framework for such evaluations. These aspects of reliability have been confirmed through various rounds of testing, with kappa values for understandability and actionability items showing moderate to strong agreement (0.40–0.84 and 0.35–0.76, respectively). Furthermore, the tool's construct validity has been bolstered through consumer testing. So, the PEMAT tool could help lay and health professionals select proper education materials that reduce health literacy demands^11^. When both the understandability score and the actionability score on the PEMAT-A/V are below 70 points, the videos might be deemed to be of low quality.

The current study showed that the YouTube tinnitus video clips created by otolaryngologists had an average score of above 70 for both categories, while both scores from the other creators were less than 70. Interestingly, the understandability score was significantly higher among otolaryngologists, reflecting their specialized knowledge that may enable them to present more clear and defined topics. In contrast, although the actionability score for otolaryngologists was higher, there was no significant difference compared to others, and both displayed relatively high scores. This might suggest that solutions provided by other medical professionals or traditional Korean medicine doctors, such as meditation, may be comparatively easier to implement (regardless of their efficacy). This could reflect a deliberate strategy to make the content more appealing to a broader audience and may be interpreted in the context of the ‘attention economy.’ It underscores the importance of being cautious when interpreting these scores, especially since high actionability may not always equate to medically sound advice.

In addition to the PEMAT-A/V, the modified DISCERN tool was used to assess the quality of medical information in the videos. This tool has been widely employed in various studies for the evaluation of medical video quality and has shown correlations with other quality assessment tools such as the Journal of American Medical Association (JAMA) score, and Global Quality Score (GQS)^9,12,13^. Our findings indicated relatively lower modified DISCERN scores for video clips produced by non-otolaryngologists, or 'others', with particular deficits observed in the reliability and reference sections of the assessment. This implies an increased likelihood of the dissemination of inaccurate information when the content is created by individuals other than otolaryngologists. It is noteworthy that this group includes not only traditional Korean medicine doctors but also other medical professionals such as non-otolaryngologist physicians, dentists, pharmacists, and physical therapists. They were found to actively propagate unproven treatments, such as nutritional supplements and vitamin intake. This underscores the potential harm associated with videos uploaded by these professionals.

The popularity and quality of YouTube videos on tinnitus were found to be unrelated in the present study. Upon examining the titles of top-viewed videos, it was found that videos promoting cheap and simple treatments such as acupressure and meditation ranked higher, most of which were not medically validated treatments. YouTube's default algorithm for video arrangement is based on several metrics, including the number of views, interaction index, and viewing rate. Especially in medical videos, recent systematic review concluded that YouTube algorithm for video recommendation could lead users to problematic contents^26^. In order to attract viewership for tinnitus-related YouTube videos that convey accurate information, a strategic approach is necessary. This would involve understanding the YouTube recommendation algorithm and selecting ideal video titles to capture viewers' attention, as well as utilizing other social media platforms for promotion. A concerted effort is needed to promote high-quality videos produced by tinnitus experts.

The present study represents the first investigation into tinnitus-related content on YouTube in the Korean language. However, the limitation of the study including the use of cross-sectional analysis, which prevented confirmation of time-series changes, such as the number of video subscriptions or number of views. Furthermore, a lack of detailed metadata, including age, gender, and regional distribution of viewers, limited the ability to conduct a more thorough and nuanced analysis. To add on, as the videos analyzed were limited to the Korean language only, this may limit the generalizability of our findings. Additionally, there may have been potential bias in the video search process, as no search query is perfect and our results are based on the specific search terms we used. Lastly, our methodological approach of only analyzing the top 100 videos might have overlooked other relevant content. Despite these limitations, this study provides important insights into the current state of tinnitus-related content on YouTube in the Korean language and highlights the need for improved regulation and guidance for content creators, as well as the provision of accurate and reliable medical information for viewers.

In conclusion, otolaryngologists should be aware of the potential impact of these videos on patient education and consider creating their own reliable and accurate content for proper understanding of tinnitus. In addition, efforts must be made to enhance the visibility and accessibility of scientific medical information on popular social media platforms.

## Data Availability

This study did not involve the use of medical data or patients' information, and all data analyzed in this study were obtained from publicly available sources. The dataset used for this study can be directly accessed via YouTube (https://www.youtube.com/).
